# Effect of COVID-19 Disease on Serum Vitamin D Status in Children with Asthma—A Retrospective Study

**DOI:** 10.3390/jcm14134525

**Published:** 2025-06-26

**Authors:** Jaqueline Abdul-Razzak, Mihaela Ionescu, Radu Diaconu, Alexandru Dan Popescu, Elena Carmen Niculescu, Ileana Octavia Petrescu, Cristina Elena Singer, Lucrețiu Radu, Liliana Anghelina, Cristian Gheonea

**Affiliations:** 1 Doctoral School, University of Medicine and Pharmacy of Craiova, 200349 Craiova, Romania; jaquelineabdulrazzak90@gmail.com; 2 Department of Pediatrics “Mother and Child”, Faculty of Medicine, University of Medicine and Pharmacy of Craiova, 200349 Craiova, Romania; carmen.niculescu@umfcv.ro (E.C.N.); ileana.petrescu@umfcv.ro (I.O.P.); cristina.singer@umfcv.ro (C.E.S.); liliana.anghelina@umfcv.ro (L.A.); cristian.gheonea@umfcv.ro (C.G.); 3 Department of Medical Informatics, Faculty of Dental Medicine, University of Medicine and Pharmacy of Craiova, 200349 Craiova, Romania; 4 Department of Endodontics, Faculty of Dental Medicine, University of Medicine and Pharmacy of Craiova, 200349 Craiova, Romania; alexandrudanpopescu20@gmail.com; 5 Department of Hygiene, Faculty of Medicine, University of Medicine and Pharmacy of Craiova, 200349 Craiova, Romania; lucretiu.radu@gmail.com

**Keywords:** pediatric asthma, COVID-19, vitamin D, FeNO, pulmonary function test, acute respiratory infection

## Abstract

**Background/Objectives**: Vitamin D is known to decrease the risk of contracting respiratory infections and developing exacerbations for children with asthma. This research evaluates the alterations in serum vitamin D concentrations and examines lung function in children with asthma, as indicated by clinical symptoms and paraclinical results, after experiencing SARS-CoV-2 infection or other acute respiratory infections. **Material and Method**: This retrospective study included 145 children with asthma. For each patient, the following variables were acquired: demographic data, serum vitamin D levels, GINA asthma control levels, the fraction of exhaled nitric oxide (FeNO), pulmonary function tests parameters, data related to allergies, and the presence of exacerbations. Children were divided into two groups, according to the presence or absence of SARS-CoV-2 infection or other acute respiratory infections. Variables were statistically processed in SPSS. **Results**: In total, 93 children with asthma with SARS-CoV-2 infection or other acute respiratory infections and 52 children with asthma without SARS-CoV-2 infection or other acute respiratory infections were included in the study. Median serum vitamin D values were statistically significantly lower in children with a variable airflow limitation, compared to children with normal values (*p* = 0.004), as well as for children with partially controlled asthma, relative to children with well controlled asthma (*p* < 0.0005). Similarly, children with acute respiratory infections/COVID-19 disease had lower median values of serum vitamin D, compared to children without acute respiratory infections/COVID-19 disease (*p* < 0.0005). A decrease in serum vitamin D value was statistically significantly associated with an increase in FeNO value for children with asthma with COVID-19 disease (*p* = 0.027), as well as for the entire study group (*p* < 0.0005). **Conclusions**: Children with asthma who had acute respiratory infections, including COVID-19 disease, showed considerably reduced serum vitamin D levels and were linked to more significant airflow limitation, reduced asthma control and elevated airway inflammation, suggesting its potential role in influencing asthma severity and infection response.

## 1. Introduction

The prevalence of asthma in pediatric age is continuously increasing in recent years and in order to achieve a good control of this pathology it is necessary to have a correct diagnosis, an appropriate placement within the treatment step and a good understanding of the disease by children and parents [1,2]. There are a variety of factors that may cause exacerbations, including acute respiratory infections, aeroallergens, cigarette smoke or exercise [3].

The pathophysiologic process in asthma is the inflammation of the airways with the reduction of their diameter, leading to the activation of dendritic cells, eosinophils, neutrophils, mast cells, lymphocytes and various cytokines, which are responsible for the development of asthma features such as increased mucus secretion and bronchial hyperreactivity. Therefore, the treatment of asthma is to relieve the associated symptoms by reducing inflammation, which would limit the risk of exacerbations [4,5].

Vitamin D is known for its anti-inflammatory property, its serum concentrations playing an important role in shaping innate or acquired immunity. A vitamin D value > 30 ng/mL will result in a significant decrease in inflammatory cytokines; lower values (<15 ng/mL) do not have the ability to inhibit the inflammatory process [6,7]. Children with asthma who receive a dose of 400–1000 IU/day of vitamin D have a lower risk of contracting respiratory infections and developing exacerbations [8,9]. Vitamin D is a metabolite accessible to the general population, with numerous benefits for the body, being considered a secosteroid, having a similar structure to estrogen, testosterone, mineralocorticoids and glucocorticoids, thus potentiating its anti-inflammatory value [8,9,10,11,12,13,14,15].

The SARS-CoV-2 virus primarily affects the airways of the respiratory tract causing symptoms such as dry cough, dyspnea and other general signs such as fever, wheezing, dizziness, taste disorders or digestive manifestations. Children with chronic conditions such as heart or lung disease are vulnerable to contracting COVID-19 disease and are also at risk of developing multisystem inflammatory syndrome (MIS) 3–4 weeks after SARS-CoV-2 infection [16,17,18,19,20]. Additionally, patients who were infected with SARS-CoV-2 showed low vitamin D values, suggesting the benefit of vitamin D administration to provide a better prognosis, not only in COVID-19 disease but also in other respiratory infections [21].

SARS-CoV-2 infection can have negative consequences on patients with chronic diseases, in the case of asthma, an insufficient level of vitamin D can cause an increase in the frequency of exacerbations, as well as changes in lung function. As vitamin D has cytokine cascade inhibitory properties, it is essential to maintain an optimal serum level and even to supplement it in special epidemiologic conditions, such as COVID-19 pandemic, thus helping patients at risk [22,23,24,25,26].

The literature includes data on how vitamin D levels affected adult patients who had COVID-19 disease and who also had other associated chronic diseases. However, given the small number of SARS-CoV-2 infections in the pediatric population and the mild to moderate symptoms they have presented, few studies have been conducted to show the consequences of SARS-CoV-2 infection, especially in children with asthma. The novelty of this study is the correlation of serum vitamin D values with pulmonary function parameters and with nitric oxide in the exhaled air of children with asthma with COVID-19 disease or other acute respiratory infections. The hypothesis of this study is that there is an association between serum vitamin D levels and FeNO values along with pulmonary function parameters, for this category of patients.

The aim of this study is to assess changes in serum vitamin D levels and to evaluate lung function in children with asthma expressed through clinical symptoms and paraclinical findings following SARS-CoV-2 infection or other acute respiratory infections.

## 2. Materials and Methods

### 2.1. Study Design and Participants’ Selection

In this retrospective study, it was analyzed the level of serum vitamin D in a group of children who were previously diagnosed with asthma and who were infected with the SARS-CoV-2 virus or had other acute respiratory infections.

For this study, the following data were collected from each subject: demographic data, data related to allergies and asthma phenotype, data regarding the presence of exacerbations, treatment steps, as well as the GINA asthma control levels [27]. It also included values regarding the fraction of exhaled nitric oxide (FeNO) and the pulmonary function tests parameters. The SARS-CoV-2 infections and other acute respiratory infections were evaluated in terms of the level of serum vitamin D and how its values interfered with the outcome of children with asthma over time.

The level of serum vitamin D was measured for each child with asthma included in the study 3–4 weeks after COVID-19 disease or after an acute respiratory infection. Serum vitamin D levels were measured using the Alinity i 25-hydroxyvitamin D (25-OH Vitamin D) Immunoassay Reagent Kit (Abbott, Longford, Ireland) using the CMIA (chemiluminescent microparticle immunoassay) method. The range values of serum vitamin D for pediatric population are as follows: sufficiency ≥ 30 ng/mL, insufficiency between 20 and 29 ng/mL, and deficiency < 20 ng/mL.

To determine the lung function of children with asthma, the following parameters such as FVC (forced vital capacity), FEV1 (forced expiratory volume in the first second), PEF (peak expiratory flow) and FEF_25–75_ (forced mid expiratory flow) were recorded. It was also documented the airflow limitation using FEV1/FVC ratio (normal range values for FEV1/FVC ratio for pediatric population is >0.9 according to GINA guideline) [27]. The diagnostic device used to measure these pulmonary function tests parameters was the Vitalograph Pneumotrac 6800 spirometer (Vitalograph, Hamburg, Germany).

To measure FeNO values, the Aerocrine Niox Vero 12-1000 analyzer (NIOX Group plc, Uppsala, Sweden) was used, the results of this test being expressed in ppb (parts per billion).

SARS-CoV-2 infection was confirmed using a PCR test or a rapid antigen test. Other acute respiratory infections included in the study were Influenza A, Influenza B and Respiratory Syncytial Virus, confirmed by rapid antigen tests.

The study included children diagnosed with asthma in whom serum vitamin D levels were collected and who benefited from periodic follow-ups within the pediatric department of a regional tertiary hospital (Filantropia Clinical Municipal Hospital Craiova, Romania) during the COVID-19 pandemic and in the post-pandemic period (March 2020–July 2024).

The inclusion criteria were as follows:

(a) Children with asthma who are less than 18 years old whose parents or legal tutors agreed to their participation in this study;
(b) Children with known asthma under treatment;
(c) Children with asthma who have measured serum vitamin D levels.

Exclusion criteria were as follows:

(a) Children with asthma who present other chronic pathologies that may intervene in the results of the present study;
(b) Children with asthma who did not have measured serum vitamin D levels.

To ensure sufficient generalizability and power for this study, the minimum number of participants was determined to be 134, value computed using the software application G*Power version 3.1.9.7, Heinrich Heine University Düsseldorf, Germany, based on a significance level α of 0.05, a power 1 − β equal to 0.8, and an effect size value of 0.5.

The study was approved by the Ethics Committee of the University of Medicine and Pharmacy of Craiova, no. 167/14.09.2023 and respected the Declaration of Helsinki. All subjects’ parents or legal tutors signed an informed consent form on behalf of pediatric patients.

### 2.2. Statistical Analysis

Study data were analyzed using SPSS (Statistical Package for Social Sciences) software, version 26 (SPSS Inc., Armonk, NY, USA). The Shapiro–Wilk test was used to evaluate the normality of continuous data series. Thus, based on the results, continuous variables were described as median values. Comparisons between various groups were performed using the Mann–Whitney U test and Kruskal–Wallis H test followed by pairwise comparisons based on Dunn’s procedure, in association with a Bonferroni correction recommended for multiple comparisons and Spearman’s rank-order correlation. Nominal and ordinal variables were defined as frequencies and percentages and were tested using the Chi-square test. The threshold of statistical significance was set to a *p*-value less than 0.05.

## 3. Results

**Figure 1:**
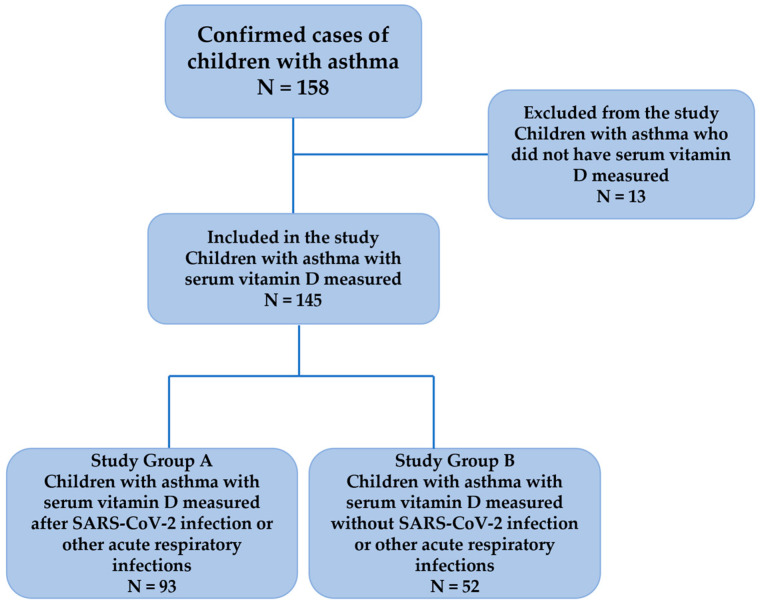
The structure of the study group.

Following the inclusion/exclusion criteria, a number of 145 children (90 boys) were finally enrolled in this study (study group A and study group B) (Figure 1).

### 3.1. Vitamin D Analysis

The study group included 145 children diagnosed with asthma, with ages less than 18 years old, and with the following gender distribution: 55 girls (37.90%) and 90 boys (62.10%). Relative to the residence area, the children from the study group mostly had an urban residence (104, 71.72%), and only less than a quarter were from rural areas (41, 28.28%). A Mann–Whitney U test was performed to identify if there were differences in serum vitamin D by gender or residence. Distributions of serum vitamin D for children within those categories were similar, as assessed by visual inspection. Median serum vitamin D value was not statistically significantly different between girls and boys, or between children from urban and rural areas, *p* > 0.05 (Table 1).

Half of the children included in the study group were diagnosed with asthma at ages between 6 and 11 years old (74 children, 51.03%). A significant percentage was dedicated to children who were diagnosed at ages less than 6 years old (44.14%), and only 4.83% (7 children) were diagnosed at ages above 12 years old. Children diagnosed at 6–11 years old had the highest serum vitamin D median value (28.50 ng/mL), followed closely by children diagnosed at early ages (27 ng/mL), while those diagnosed as teenagers exhibited the lowest median value (23 ng/mL). The result of the Mann–Whitney test regarding the presence of statistically significant differences in the serum vitamin D levels between children with various ages at diagnosis, yielded a value close to the statistical threshold, *p* = 0.074, potentially emphasizing the idea that higher ages at diagnosis could be associated with smaller values of serum vitamin D, still with caution relatively to the small number of children within the last age group (Table 1).

Serum vitamin D values were not statistically significantly different between children with and without the following allergies or allergic and atopic diseases: allergic rhinitis, atopic dermatitis, allergies regarding food, pollen, house dust, animal hair, mold or tobacco (*p* > 0.05; Table 1).

Table 2 presents the clinical context of the children included in the study lot. An analysis of serum vitamin D values by treatment step revealed that children within step 4 of treatment had a median value of 45 ng/mL, while all other children in various steps of the treatment had a median value smaller than or equal to 28 ng/mL. Still, there were no statistically significant differences in the serum vitamin D levels between these groups, *p* > 0.05. In relation to the asthma phenotype and parents with predisposition to atopy, there were no statistically significant differences between children from these groups, *p* > 0.05.

A Mann–Whitney U test was run to determine the differences in serum vitamin D values between children with a normal FEV1/FVC ratio or variable airflow limitation. Distributions of serum vitamin D for children within both categories of FEV1/FVC ratio were similar, as assessed by visual inspection. Median serum vitamin D value was statistically significantly lower in children with a variable airflow limitation (26 ng/mL) than in children with a normal value of FEV1/FVC ratio (36 ng/mL), *p* = 0.004. Children with partially controlled asthma have statistically significantly lower median serum vitamin D values (24 ng/mL), compared to children with well-controlled asthma (37 ng/mL), *p* < 0.0005. Similarly, children with acute respiratory infections/COVID-19 disease have lower median values of serum vitamin D (23 ng/mL), compared to children without acute respiratory infections/COVID-19 disease (46.50 ng/mL), and the differences between groups are statistically significant, *p* < 0.0005 (Table 2).

A Kruskal–Wallis test was performed to determine the differences in serum vitamin D levels between groups that differed in number of exacerbations per year: children with no exacerbation (*n* = 51), children with 1 exacerbation (*n* = 56), children with 2 exacerbations (*n* = 30) and children with 3 exacerbations (*n* = 8). Distributions of serum vitamin D values were similar for all groups, as assessed by visual inspection of a boxplot. Children with asthma with no exacerbations per year had a high median value of serum vitamin D, compared to children with at least one exacerbation per year; in fact, the median values of serum vitamin D decrease as the number of exacerbations per year increases. Median serum vitamin D values were statistically significantly different between children with different numbers of exacerbations, χ^2^(3) = 34.844, *p* < 0.0005. Subsequently, pairwise comparisons were completed using Dunn’s procedure. A Bonferroni correction for multiple comparisons was applied, with the statistical significance accepted for *p* < 0.0083. The post hoc analysis revealed statistically significant differences in serum vitamin D values between children with two exacerbations (21 ng/mL) and children with one exacerbation (26 ng/mL) (*p* = 0.005), and children with three exacerbations (13.50 ng/mL) (*p* < 0.0005) and children with no exacerbation (46 ng/mL) (*p* < 0.0005), but not between any other group combination (Table 2).

A Spearman’s rank-order correlation was performed to assess the potential association between serum vitamin D and FeNO, both for children with COVID-19 disease, and the entire study lot. The preliminary analysis determined the relationship to be monotonic, as assessed by visual inspection of a scatterplot. There was a statistically significant, moderate negative correlation between these variables, a decrease in serum vitamin D value being associated with an increase in the FeNO value, rs(77) = −0.249, *p* = 0.027 for children with COVID-19 disease, and rs(143) = −0.476, *p* < 0.0005 for the entire study group. Similar correlations were analyzed for pulmonary function tests parameters, FVC, FEV1, PEF, FEF_25–75_ and FEV1/FVC ratio, but no statistically significant associations were identified, *p* > 0.05 (Table 3).

### 3.2. Acute Respiratory Infections Analysis

Almost two thirds of children included in the study group presented acute respiratory infections (93 children, 64.14%). As described in Table 4, there are no statistically significant associations between acute respiratory infections and gender, residence and age at asthma diagnosis.

According to Table 5, the presence of acute respiratory infections is similarly distributed among children with asthma with various numbers of exacerbations per year, no association could be identified between these parameters, *p* > 0.05. Similar results are obtained regarding the presence of an atopic disease for at least one of the parents, *p* > 0.05.

Statistically significant associations were identified for acute respiratory infections and the FEV1/FVC ratio, GINA asthma control levels, and asthma phenotype. Around 80% of children with a variable airflow limitation have acute respiratory infections, compared to 35.42% children with a normal FEV1/FVC ratio. Similarly, 63.44% of children with partially controlled asthma have acute respiratory infections, compared to only 36.56% of children with well-controlled asthma. Asthma phenotype is also associated with acute respiratory infections, as 75% of children with a non-allergic phenotype have acute respiratory infections, compared to only 57.30% of children with an allergic phenotype (Table 5).

### 3.3. COVID-19 Analysis

A previous infection with SARS-CoV-2 virus was recorded for more than half of children included in the study group (79 children, 54.48% group). A Mann–Whitney U test was run to determine if there were differences in serum vitamin D between children with and without COVID-19 disease. Distributions of serum vitamin D for children within both categories were similar, as assessed by visual inspection. Median serum vitamin D value was statistically significantly lower in children with a previous SARS-CoV-2 infection (24 ng/mL) than in children without a previous infection (44 ng/mL), *p* < 0.0005 (Table 6).

## 4. Discussion

The COVID-19 pandemic caused changes in people’s daily routines, with the pediatric population suffering from chronic pathologies, being a category that required additional attention during this period [28]. The SARS-CoV-2 virus has shown a rather high level of contagiousness, ranging from mild symptoms to complications such as acute respiratory distress syndrome (ARDS), affecting both children and adults. Asthma, with an increasing number of cases among pediatric patients, has been a cause of concern for physicians worldwide [29,30,31].

In the present study, differences in serum vitamin D values were noted, which influenced the clinical and paraclinical outcomes of children with asthma during the COVID-19 pandemic and post-pandemic period following contract with SARS-CoV-2 infection or other acute respiratory infections compared to children with asthma who did not contract COVID-19 disease or other acute respiratory infections.

Recent studies have observed a significant decrease in airway inflammation and bronchial hyperresponsiveness leading to a decrease in the number of exacerbations, especially those induced by acute respiratory infections, with a standard dose of vitamin D given daily for 6 months producing these benefits [32,33,34,35]. In a study by Fedora et al., it was found that patients who had a lower incidence of exacerbations were those who were adherent to corticosteroids and vitamin D, thus proving the synergistic effects that vitamin D has with asthma treatment, especially in children [36].

Vitamin D acts through the vitamin D receptor (VDR) being expressed in approximately all immune cells in the body such as dendritic cells and macrophages, playing a role in improving the antimicrobial properties of these cells and therefore reducing viral replication and the production of anti-inflammatory cytokines [37,38,39,40].

Infection with SARS-CoV-2 virus causes an amplification of inflammatory cytokines (IL-6 and IFN-γ being expressed in children), which are in fact immune cells that are directed to the site of infection affecting the lungs through tissue destruction [41,42,43]. Recent studies have shown that a low level of vitamin D in children is a risk factor for COVID-19 disease and therefore an unfavorable prognosis of this infection, also being a predictor of an eventual MIS-C (Multisystemic inflammatory Syndrome in Children) [44,45,46,47].

In the current study, the number of exacerbations in children with asthma was inversely related to the serum vitamin D value, so that the lower the serum vitamin D value, the higher the number of exacerbations. Likewise, low serum vitamin D values were observed in children with asthma who had acute respiratory infections including SARS-CoV-2 infection.

Pro-inflammatory cells, such as Il-7 and Il-3, are involved in the pathogenesis of asthma, along with the increase in Th-17 cells, causing an imbalance between Th1 and Th2 cells. A low level of vitamin D affects the composition of the intestinal microbiome and therefore causes a low production of short-chain fatty acids (SCFAs), with a reduction in the maturation of dendritic cells and an increase in Th-17 activity. A decrease in SCFAs has consequences for the cells of the lung epithelium affected by asthma, with children with asthma being more susceptible to contract acute respiratory infections [9,48,49,50,51]. The influence of the SARS-CoV-2 virus on intestinal microbiota has also been proven, along with its effects on lung function [52,53].

The pulmonary function of children with asthma should be constantly monitored, especially under special epidemiological conditions. Studies have shown a correlation between vitamin D levels and lung function in children with asthma, so that vitamin D deficiency caused a decrease in spirometric values such as FEV1, FVC, and FEV1/FVC ratio, leading to impaired lung function. During infection with SARS-CoV-2, children with asthma show lung tissue damage, so that low values of FVC and FEV1 have been observed [54,55,56,57,58,59,60,61,62]. In the present study, children with asthma with COVID-19 disease or other acute respiratory infections had low serum vitamin D values and were more susceptible to pulmonary function changes and thus to variable airflow limitation.

Another parameter that was altered is nitric oxide in exhaled air. Children with asthma with low vitamin D levels showed elevated FeNO values, indicating airway inflammation compared to pediatric populations with asthma whose normal vitamin D values did not influence lung function [63,64]. The current research reported a statistically significant correlation between low serum vitamin D levels and elevated FeNO values.

Regarding the level of asthma control, studies have highlighted the importance of vitamin D in children with asthma and how this fat-soluble vitamin influences the course of chronic lung disease. Low serum vitamin D levels have been observed in patients with partially controlled and uncontrolled asthma, which may increase the risk of exacerbations [65,66]. This was also emphasized in the present study, children with asthma who had low serum vitamin D values were more likely to develop a more difficult disease course with exacerbations, which was observed in patients with partially controlled asthma (24 ng/mL) compared to patients with asthma whose normal serum vitamin D values had well controlled asthma (37 ng/mL).

Vitamin D has numerous benefits on the organism not only in preventing rickets but also in preventing contact with various respiratory infections, being also a predictor of acute respiratory infections in patients with asthma, avoiding exacerbations and determining a better control of lung function, thus improving the quality of life of pediatric patients [67,68,69]. The administration of a daily maintenance dose of vitamin D offers superior benefits compared to bolus administration of vitamin D weekly or every 1–3 months, thus keeping serum vitamin D levels within normal parameters and preventing the risk of acute respiratory infections, including SARS-CoV-2 infection [9,70,71,72,73].

Other micronutrients, such as B-complex vitamins, are also known to be critical for an effective immune response to viral pathogens, including SARS-CoV-2 infection. Recent studies have indicated that B vitamins are linked to better prognosis in COVID-19 outcomes. Thus, a holistic nutritional strategy, ensuring sufficiency of both vitamin D and other key immunomodulatory nutrients, may be the most effective approach for supporting respiratory health [53,74].

In this research, it can be observed that children with asthma with COVID-19 disease had low serum vitamin D values causing inflammatory changes in the airways observed both clinically and paraclinically. However, children with asthma who had other acute respiratory infections or who had not contracted SARS-CoV-2 infection had higher serum vitamin D values compared to those who had contracted COVID-19 disease, suggesting the importance of vitamin D in children with asthma, an optimal serum level being beneficial in special epidemiologic conditions, avoiding an unfavorable course of the disease.

### 4.1. Limitations of the Study

Due to the fact that the study period coincided with the COVID-19 pandemic period, children with asthma did not present for routine check-ups unless absolutely necessary, i.e., in case of medical emergencies, making it difficult to collect medical data and to assess the evolution of the disease over time. Another limitation of the study is that the number of children with asthma for whom the measured serum vitamin D was reduced because parents/legal guardians wanted to avoid the risk of contracting acute respiratory infections and thus SARS-CoV-2 infection during the COVID-19 pandemic. Additionally, due to the lock-down, access to routine medical services was limited, with major medical emergencies being prioritized and low vitamin D levels could be a consequence of limited outdoor activity and reduced sun exposure in children with asthma.

### 4.2. Recommendations for Further Research

This study provides valuable information on the effects that COVID-19 disease has on serum vitamin D levels in children with asthma. To consolidate the results of this study and better understand the impact of acute respiratory infections on children with asthma, additional research is needed to monitor the evolution of lung function, FeNO and serum vitamin D status. Additionally, including vitamin D dosing in the monitoring of children with asthma and evaluating FeNO as a potential predictive marker of airway inflammation could contribute to an individualized treatment. Further research is required to involve a wider population of children with asthma and time monitoring of serum vitamin D values to highlight the impact that SARS-CoV-2 infection had on children with asthma.

## 5. Conclusions

Children with asthma and acute respiratory infections, including COVID-19 disease, had significantly lower serum vitamin D levels, require a personalized therapeutic approach, especially in specific epidemiological conditions such as the COVID-19 pandemic. Lower serum vitamin D levels were associated with worse airflow limitation, poorer asthma control, and increased airway inflammation, highlighting its potential role in asthma severity and infection response.

A key finding is the significant negative correlation between serum vitamin D levels and FeNO values, a marker of airway inflammation, after COVID-19 disease. This retrospective study highlights certain aspects regarding the quality of asthma management in children such as serum vitamin D dosing along with supplementation, when necessary, implementation and compliance with an individualized therapeutic plan and constant medical follow-ups. All of these could be considered essential strategies for better and lasting control of the disease.

## Figures and Tables

**Table 1:** Distribution of the study group according to demographic data, allergic and atopic diseases.

Parameter	Category	145 Children	Vitamin D	*p* *
			Median Value (ng/mL)	
Gender	Females	55 (37.90%)	27	0.217
	Males	90 (62.10%)	28	
Environment	Urban	104 (71.72%)	27	0.855
	Rural	41 (28.28%)	28	
Age at asthma diagnosis	<6 years old	64 (44.14%)	27	0.074
	6–11 years old	74 (51.03%)	28.50	
	12–17 years old	7 (4.83%)	23	
Allergic rhinitis	Present	90 (62.07%)	28	0.626
	Absent	55 (37.93%)	27	
Atopic dermatitis	Present	23 (16.08%)	27	0.697
	Absent	120 (83.92%)	28	
Allergies regarding food	Present	13 (8.97%)	29	0.898
	Absent	132 (91.03%)	27	
Pollen	Present	27 (18.62%)	28	0.865
	Absent	118 (81.38%)	27	
House dust	Present	32 (22.07%)	24	0.101
	Absent	113 (77.93%)	28	
Animal hair	Present	20 (13.79%)	25.50	0.372
	Absent	125 (86.21%)	28	
Mold	Present	23 (15.86%)	24	0.105
	Absent	122 (84.14%)	28	
Tobacco	Present	3 (2.07%)	25	0.257
	Absent	142 (97.93%)	27.50	

* Mann–Whitney test.

**Table 2:** Distribution of the study group according to serum vitamin D and different variables.

Parameter	Category	145 Children	Vitamin D	*p*
			Median Value (ng/mL)	
Treatment step	Step 1	5 (3.45%)	28	0.415 *
	Step 2	88 (60.69%)	28	
	Step 3	46 (31.72%)	25.50	
	Step 4	5 (3.45%)	45	
	Step 5	1 (0.69%)	8	
FEV1/FVC ratio	Normal	48 (33.10%)	36	0.004 **
	Variable airflow limitation	97 (66.90%)	26	
Exacerbations	0/year	51 (35.17%)	46	<0.0005 *^,#^
	1/year	56 (38.62%)	26	
	2/year	30 (20.69%)	21	
	3/year	8 (5.52%)	13.50	
GINA asthma control levels	Well controlled	71 (48.97%)	37	<0.0005 **^,#^
	Partially controlled	74 (51.03%)	24	
Asthma Phenotype	Allergic	89 (61.38%)	28	0.572 **
	Non-allergic	56 (38.62%)	27	
Parents with predisposition to atopy	Yes	34 (23.45%)	28.50	0.437 **
	No	111 (76.55%)	27	
Acute respiratory infections/COVID-19 disease	Yes	93 (64.14%)	23	<0.0005 **^,#^
	No	52 (35.86%)	46.50	

* Kruskal–Wallis test. ** Mann–Whitney test. ^#^ Statistically significant. FEV1—Forced Expiratory Volume in the First Second. FVC—Forced Vital Capacity.

**Table 3:** Associations between pulmonary function tests parameters and serum vitamin D for the study group.

Parameter	Vitamin D			
	COVID-19 (*n* = 79)		Study Group (*n* = 145)	
	Correlation Coefficient	*p* *	Correlation Coefficient	*p* *
FeNO	−0.249	0.027	−0.476	<0.0005 ^#^
FVC	0.046	0.690	0.032	0.701
FEV1	−0.007	0.948	0.113	0.176
PEF	0.027	0.811	−0.043	0.610
FEF_25–75_	0.036	0.755	0.053	0.523
FEV1/FVC ratio	−0.190	0.094	0.105	0.210

* Spearman’s rank-order correlation. ^#^ Statistically significant. FeNO—Fraction of Exhaled Nitric Oxide. FVC—Forced Vital Capacity. FEV1—Forced Expiratory Volume in the First Second. PEF—Peak Expiratory Flow. FEF_25–75_—Forced Mid Expiratory Flow.

**Table 4:** Distribution of the study group by the presence of acute respiratory infections and demographic data.

**Parameter**	**Category**	**Acute Respiratory Infections**			*p* *
		**Yes**	**No**	**Total**	
		93 (64.14%)	52 (35.86%)	145 (100%)	
Gender	Females	37 (67.27%)	18 (32.73%)	55 (100%)	0.538
		39.78%	34.62%		
	Males	56 (62.22%)	34 (37.78%)	90 (100%)	
		60.22%	65.38%		
Environment	Urban	66 (63.46%)	38 (36.54%)	104 (100%)	0.787
		70.97%	73.08%		
	Rural	27 (65.85%)	14 (34.15%)	41 (100%)	
		29.03%	26.92%		
Age at asthma diagnosis	<6	38 (56.72%)	29 (43.28%)	67 (100%)	0.844
		46.91%	42.65%		
	6–11	39 (52%)	36 (48%)	75 (100%)	
		48.15%	52.94%		
	12–17	4 (57.14%)	3 (42.86%)	7 (100%)	
		4.94%	4.41%		

* Chi-square test. The values in grey are summed by columns.

**Table 5:** Distribution of the study group according to acute respiratory infections and different variables.

**Parameter**	**Category**	**Acute Respiratory Infections**			*p* *
		**Yes**	**No**	**Total**	
		93 (64.14%)	52 (35.86%)	145 (100%)	
Exacerbations	0/year	16 (31.37%)	35 (68.63%)	51 (100%)	0.110
		17.2%	67.31%		
	1/year	47 (83.93%)	9 (16.07%)	56 (100%)	
		50.54%	17.31%		
	2/year	23 (76.67%)	7 (23.33%)	30 (100%)	
		24.73%	13.46%		
	3/year	7 (87.5%)	1 (12.5%)	8 (100%)	
		7.53%	1.92%		
FEV1/FVC ratio	Normal	17 (35.42%)	31 (64.58%)	48 (100%)	<0.0005 ^#^
		18.28%	59.62%		
	Variable airflow limitation	76 (78.35%)	21 (21.65%)	97 (100%)	
		81.72%	40.38%		
GINA asthma control levels	Well controlled	34 (47.89%)	37 (52.11%)	71 (100%)	<0.0005 ^#^
		36.56%	71.15%		
	Partially controlled	59 (79.73%)	15 (20.27%)	74 (100%)	
		63.44%	28.85%		
Asthma phenotype	Allergic	51 (57.30%)	38 (42.70%)	89 (100%)	0.031 ^#^
		54.84%	73.08%		
	Non-allergic	42 (75%)	14 (25%)	56 (100%)	
		45.16%	26.92%		
Parents with predisposition to atopy	Yes	20 (58.82%)	14 (41.18%)	34 (100%)	0.460
		21.51%	26.92%		
	No	73 (65.77%)	38 (34.23%)	111 (100%)	
		78.49%	73.08%		

* Chi-square test. ^#^ Statistically significant. The values in grey are summed by columns. FEV1—Forced Expiratory Volume in the First Second. FVC—Forced Vital Capacity.

**Table 6:** Serum vitamin D values distribution based on COVID-19 disease presence and the presence of acute respiratory infections.

**Parameter**	**Category**	**COVID-19**			*p* *
		**Yes**	**No**	**Total**	
		79 (54.48%)	66 (45.52%)	145 (100%)	
Vitamin D	Median values (ng/mL)	24	44	-	<0.0005 *^,#^
Acute respiratory infections	Yes	79 (84.95%)	14 (15.05%)	93 (100%)	<0.0005 **^,#^
		100%	21.21%		
	No	0 (0%)	52 (100%)	52 (100%)	
		0%	78.79%		

* Mann–Whitney test. ** Chi-square test. ^#^ Statistically significant. The values in grey are summed by columns.

## Data Availability

The authors declare that the data of this research are available from the corresponding authors upon reasonable request.

## Abbreviations

The following abbreviations are used in this manuscript:

FeNO	Fraction of Exhaled Nitric Oxide
FVC	Forced Vital Capacity
FEV1	Forced Expiratory Volume in the First Second
PEF	Peak Expiratory Flow
FEF_25–75_	Forced Mid Expiratory Flow
